# Quantification of Antiphospholipid Antibodies: The Importance of Using an Appropriate Methodology for Each Clinical Profile

**DOI:** 10.3390/ijms242417373

**Published:** 2023-12-12

**Authors:** Oscar Cabrera-Marante, Sara Garcinuño, Daniel Enrique Pleguezuelo, Francisco J. Gil-Etayo, Iulian Tenica, Edgard Rodríguez de Frías, Denis Zafra, Nerea Castro, Estela Paz-Artal, Antonio Serrano, Manuel Serrano

**Affiliations:** 1 Department of Immunology, Hospital Universitario 12 de Octubre, 28041 Madrid, Spain; oscar.cabrera@salud.madrid.org (O.C.-M.); dpleguezuelo@salud.madrid.org (D.E.P.); javier.gil.etayo@gmail.com (F.J.G.-E.); erodriguezfrias@gmail.com (E.R.d.F.); estela.paz@salud.madrid.org (E.P.-A.); mserranobl@gmail.com (M.S.); 2 Instituto de Investigación Sanitaria Hospital 12 de Octubre (imas12), 28041 Madrid, Spain; garcinunosara@gmail.com; 3 Department of Haematology, Hospital Universitario de Salamanca, 37007 Salamanca, Spain; 4 Department of Occupational Medicine, Hospital Universitario 12 de Octubre, 28041 Madrid, Spain; tenica.iulian@yahoo.com; 5 Department of Haematology, Hospital Universitario 12 de Octubre, 28041 Madrid, Spain; denis.zafra@salud.madrid.org (D.Z.); nerea.castro@salud.madrid.org (N.C.); 6 Biomedical Research Centre Network for Epidemiology and Public Health (CIBERESP), 28029 Madrid, Spain

**Keywords:** antiphospholipid syndrome, antiphospholipid antibodies, laboratory automation, autoimmune diseases

## Abstract

The presence of antiphospholipid antibodies (aPLs) is associated with antiphospholipid syndrome (APS), characterized by thrombosis and obstetric morbidity. aPLs included in APS classification criteria are lupus anticoagulant, anti-cardiolipin and anti-beta-2-glycoprotein-I of IgG or IgM isotypes. Enzyme-linked immunosorbent assay is the most used diagnostic technique to determine aPLs. Recently, new automated technologies mainly based in antigen-coated beads have been developed. The aim is to compare a fluorescence enzyme immunoassay (M1) and an antigen-coated bead assay (M2) in obstetric and thrombotic APS patients. All samples from the first 1020 patients received in the Immune Service Laboratory (Hospital 12 de Octubre) during the recruitment period, without exclusions, were analysed for aPLs. The weighted kappa for both methods in all the patients was 0.39 (0.30–0.47). Agreement increased to 0.56 (0.38–0.73) in patients with autoimmune disease. Sensitivity and specificity obtained for M1 were 17.1% and 89.3%, respectively, and 12.7% and 91.4% for M2. The sensibility and specificity of IgG isotypes were higher than the IgM ones. Regarding obstetric patients, M1 obtained significant diagnostic performance and had more sensitivity 23.75 (14.95–34.58) compared to M2 12.50 (6.16–21.79). In conclusion, clinical suspicion-based method selection for aPLs should be considered. To identify obstetric APS patients, solid phase methods remain more preferable.

## 1. Introduction

Antiphospholipid syndrome (APS) is an autoimmune disease characterized by a history of thrombosis and/or obstetric morbidities in patients carrying antiphospholipid antibodies (aPLs). Nowadays, two clinically differentiated APS sub-entities have been established: thrombotic and obstetric APS [1]. According to the 2023 ACR/EULAR Antiphospholipid Syndrome Classification Criteria, patients can be classified as APS for research purposes if they score at least three points from clinical domains and at least three points from laboratory domains [2]. Laboratory criteria include the presence of lupus anticoagulant (LA) or aPLs: anticardiolipin antibodies (aCL) or anti-β2 glycoprotein-I antibodies (aβ2GPI), either IgG or IgM [2]. Moreover, there are other aPLs not included in APS criteria that have been increasing in relevance in recent years, like aβ2GPI of isotype IgA [3] and anti-phosphatidylserine/prothrombin antibodies [4]. It has been proposed that the determination of these antibodies by ELISA could be comparable to the results of the functional test lupus anticoagulant but there are also studies that do not support this association; therefore, there is no unanimity in the scientific community [5,6].

In 1983, Harris et al. [7] used a solid phase immunoassay to create the first anti-cardiolipin antibody detection test, which was a significant milestone for the understanding of the APS condition. Historically, solid phase assays, primarily enzyme-linked immunosorbent fluorescent assays (ELISA), have been used to detect aPLs. However, the continued increase in demand for aPL testing in clinical laboratory services created a significant, unmet need in the field: automation of aPL testing. This led to the development of new methods [8]. Thus, alternative methods, such as semi-solid phase systems based on antigen-coated beads, were developed. However, the aforementioned diagnostic methods showed considerable variability between assays for the detection of aCLs [9,10] and aβ2GPI. Despite the low agreement found between the four commercially available immunoassays compared using a pool of aPL-positive patients, Chayoua et al. found that the presence of at least one positive aPL test was associated with obstetric morbidity and/or thrombosis, regardless of the immunoassay employed. In addition, Reber et al. described interlaboratory variability between homemade and commercial assays for the detection of aPLs. Likewise, notable discrepancies were found due to the lack of standardization in the low range of the calibration curve and the absence of consistent thresholds for the definition of positivity [11,12]. Other studies, such as that by Kaneshige et al., found better correlations by comparing two manual and three automated assays in Japan [13]. On the basis of the documented discrepancies, a clear limitation in method standardization was concluded, and the differences within the various epitopes used as test antigens are likely to be one of the root causes [14,15].

ELISA is unofficially considered the gold standard, as it is the most established and widely used method. Moreover, some patients could only be identified by using solid phase methods, according to a comparative workshop carried out in Spain which included over 40 laboratories [16]. Evaluation of which test best correlates with clinical symptoms is crucial. Thus, there is a need for the validation of new assays from an analytical and clinical point of view. Studies have shown that several analytical methods do not effectively detect aPLs [17,18]. This variability between assays increases complexity when trying to establish universal aPL cut-off values.

Therefore, in this study, we aim to analyse the diagnostic performance of two automated methods for detecting aPLs: one based on a solid fluoroenzyme immunoassay and the other on a semi-solid phase (antigen-coated beads). Here, we compare the diagnostic accuracy (agreement) of the IgG and IgM isotypes of aCLs and aβ2GPI antibodies in a real-world setting. We also aim to compare the performance of the two methods in classifying thrombotic and obstetric APS. Despite the existence of other aPLs not included in the classification criteria, in this study, we have focused on the criterion of aPLs because there is higher scientific evidence and availability of standardized kits for the detection of these antibodies.

## 2. Results

### 2.1. Study Population

We measured aCLs IgG/IgM and aβ2GPI IgG/IgM aPLs in 1020 individuals (61% female) with a mean age of 55.42 (54.24–56.59). Among them, 403 patients (39.5%) met the clinical criteria for APS classification. Within this subgroup, 80% (*n* = 329) had thrombotic APS and 20% (*n* = 80) were patients with APS-related obstetric morbidity—with six patients exhibiting both conditions simultaneously. Cardiovascular risk factors identified in the study cohort were high blood pressure, dyslipidaemia, smoking and diabetes mellitus, present in 324, 247, 239 and 151 patients, respectively. Additionally, 172 had a documented history of other autoimmune diseases. The most prevalent autoimmune disease in both subgroups was systemic lupus erythematosus followed by rheumatoid arthritis, sjögren syndrome and systemic sclerosis. The demographic and clinical characteristics of the cohort are described in Table 1.

### 2.2. Method Comparison

Of the 1020 patients, 18.1% (*n* = 185) were identified as positive for at least one of the studied autoantibodies, for either M1 or M2. M1 displayed a higher number of positive results than M2 (135 vs. 105 patients). Agreement between both methods was found in 87.3% of the measurements (including both positive and negative findings).

**Figure 1:**
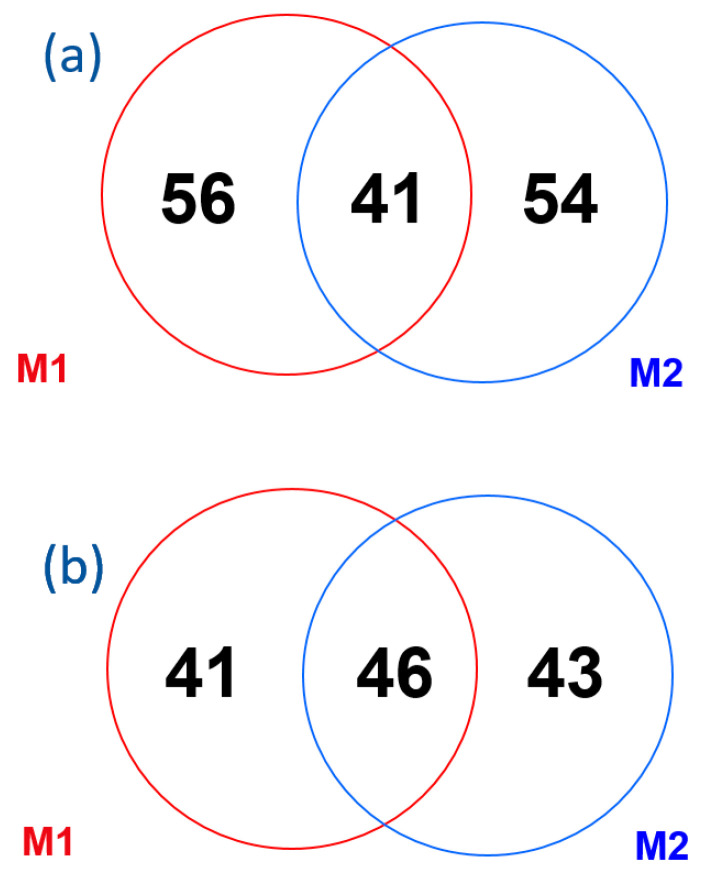
Prevalence of each aPL for the two diagnostic methods assessed in the cohort of 1020 individuals: (**a**) positive aCL antibodies of the IgG or IgM isotype, (**b**) positive aβ2GPI antibodies of the IgG or IgM isotype.

Comparing the number of positive aCLs of the IgG or IgM isotype for M1 and M2: 56 patients were positive for M1 only, 54 patients were positive for M2 only and 41 patients were positive in both methods. For aβ2GPI, it was observed that 41 patients obtained positive results for M1 only, while 43 patients achieved positive results for M2 only. Additionally, 46 patients exhibited positivity for both techniques, as depicted in Figure 1.

**Figure 2:**
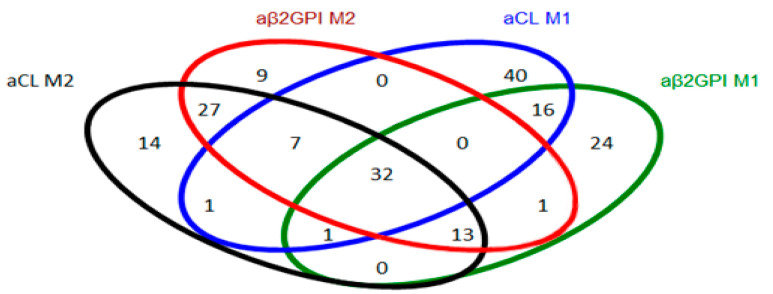
Venn diagram. Number of positive patients for each autoantibody test using both methods. A patient is considered to be positive when presenting an aPL of IgG or IgM isotype. aβ2GPI: anti-β-2 glycoprotein I autoantibodies-positive patients I; aCL: anti-cardiolipin autoantibodies-positive patients; M1: enzyme-linked immunosorbent fluorescent assay; M2, Addressable laser bead immunoassay.

Interestingly, 32 patients showed positive results for the four autoantibodies using both methods (Figure 2). M1 had the higher detection capability, displaying positive isolated results in 40 patients for aCLs. Conversely, M2 had the lower detection capability, with just nine isolated positive patients for aβ2GPI.

When individually comparing the two methods, for each antibody and isotype, the total agreement was 94.7% for both aβ2GPI IgG and IgM and also 93.8% and 92.7% for cardiolipin IgG and IgM, respectively.

The kappa index was used to evaluate the global agreement between both methods for all the patients included, yielding a value of κ = 0.38 (SD = 0.04). When analysing each autoantibody, the agreement was found to be higher for aβ2GPI in comparison to aCL (Table 2). In the case of antibodies of the IgG isotype, stronger correlations were observed as opposed to the IgM antibodies targeting the same antigen. Consequently, the aCL IgM exhibited the lowest kappa index.

**Table 2:** M1 and M2 concordance evaluation for classical antiphospholipid antibodies.

aPL	M1 and M2 Positives	M1 Positives	M2 Positives	Kappa Index	95% CI	Level of Agreement
aCL IgG	24	32	31	0.40	0.28–0.52	Fair
aCL IgM	18	35	40	0.28	0.17–0.40	Fair
aβ2GPI IgG	28	25	29	0.48	0.36–0.60	Moderate
aβ2GPI IgM	23	18	36	0.43	0.31–0.56	Moderate

Abbreviations: aPL: antiphospholipid antibody, aβ2GPI: anti-β-2 glycoprotein I-positive patients I, aCL: anti-cardiolipin-positive patients; M1: enzyme-linked immunosorbent fluorescent assay; M2, addressable laser bead immunoassay. Level of agreement calculated based on guidelines provided by Landis and Koch [19].

### 2.3. Lupus Anticoagulant

Of the 1020 patients, 549 were tested for LA, with 401 patients being negative and 148 patients being positive (26.96%). In comparing patients with a previous diagnosis of autoimmune disease and those without this kind of disorder, we observed similar proportion of LA: 29% of the patients with a previous autoimmune disease were positive for LA and also 26.5% (*p* = 0.700) of patients without this diagnosis.

We also did not find differences in the proportion of LA when we compared patients with or without APS events: 24.6% of positives in patients with clinical events and 29.4% in patients without them (*p* = 0.243).

### 2.4. Patients with APS Associated Clinical Events

Venous thrombosis was the most reported event in the 403 patients who met APS clinical criteria (*n* = 114), followed by pulmonary thromboembolism (*n* = 94), stroke (*n* = 82), obstetric morbidity (*n* = 80), myocardial infarction (*n* = 63) and arterial thrombosis (*n* = 19). All females with obstetric morbidity had three or more recurrent spontaneous miscarriages. The average age in this subgroup was 56 years, and 57.1% were females (*n* = 230). Cardiovascular risk factors were led by high blood pressure (*n* = 146) and followed by dyslipidaemia (*n* = 129), smoking (*n* = 118) and diabetes mellitus (*n* = 72). Moreover, 47 patients had been diagnosed previously with other autoimmune diseases.

### 2.5. Correlation between Methodologies According to Clinical Events

We studied, for each autoantibody and isotype, the correlation between the quantitative results obtained using both methods in patients with and without APS-related events (Table 3). The correlation coefficient was higher in patients with APS-related events than in patients without events.

In Table 4, we present the correlation between the levels of each antibody in patients who experienced thrombotic and gestational events. For both types of APS events, we observe comparable correlation coefficients.

In the sub-analysis of patients who met clinical criteria for APS, the results showed that 22.5% of them had a positive result for any of the aPL assessments: 10% of patients with obstetric APS and 6% with thrombotic APS were positive for both methods. Notably, the M1 method yielded a higher number of positive results than the M2; 11 vs. 2 positives in obstetric APS patients and 31 vs. 23 in thrombotic APS patients (Table 5).

**Table 5:** M1 and M2 concordance evaluation for classical antiphospholipid antibodies in different patient subgroups.

Clinical Manifestation	Number of Patients	M1 and M2 Positives	M1 Positives	M2 Positives	Kappa Index	95% CI	Level of Agreement
Any Sidney APS clinical criteria	403	27	42	24	0.36	0.23–0.48	Fair
Gestational only	74	7	11	1	0.46	0.21–0.70	Moderate
Gestational	80	8	11	2	0.45	0.21–0.69	Moderate
Thrombotic and gestational	6	1	0	1	0.57	−0.12–1.00	No agreement
Thrombotic only	323	19	31	22	0.32	0.18–0.46	Fair
Thrombotic	329	20	31	23	0.33	0.19–0.47	Fair

Abbreviations: APS: antiphospholipid syndrome; M1: enzyme-linked immunosorbent fluorescent assay; M2: addressable laser bead immunoassay. Level of agreement calculated based on guidelines provided by Landis and Koch [19].

The degree of statistical correlation was higher among patients who experienced obstetric events: κ = 0.46. The combination of patients suffering from both clinical manifestations decreased the correlation to κ = 0.36, which is similar to the one found in thrombotic patients: κ = 0.33 (Table 5).

As previously observed, major discrepancies were found in patients with aPL values near the cut-off value. Upon inclusion of patients with clinical manifestations and associated titres above 40 units (high positives), for at least one method, the kappa index increased from 0.36 to 0.53.

### 2.6. Patients with Other Autoimmune Disease

Of the 1020 patients included in the study, 172 (16.9%) had a pre-existing diagnosis of autoimmune diseases. Within the subgroup of 403 patients with APS-associated clinical symptoms, only 47 (11.7%) also had other autoimmune diseases.

The prevalence of APS events in patients with systemic autoimmune rheumatic diseases (SARDs) was significantly lower when compared to those with such conditions (OR: 0.52 95% CI: 0.36–0.75).

The weighted kappa by considering the positivity of any of the tests or isotypes was 0.53 (95% CI: 0.36–0.71) in patients with a previous autoimmune diagnosis. Conversely, in patients without an autoimmune disease, the weighted kappa was 0.34 (95% CI: 0.24–0.43).

The level of agreement for M1 and M2 in patients with a previous documented history of autoimmune disease and an APS-related clinical event was the highest within the scope of the study: 0.55 (95% CI: 0.28–0.83). Although not significantly, a tendency can be seen that patients with an autoimmune disease have a higher kappa index than those without autoimmune disease. See Table 6.

### 2.7. Importance of Antibody Titres

**Figure 3:**
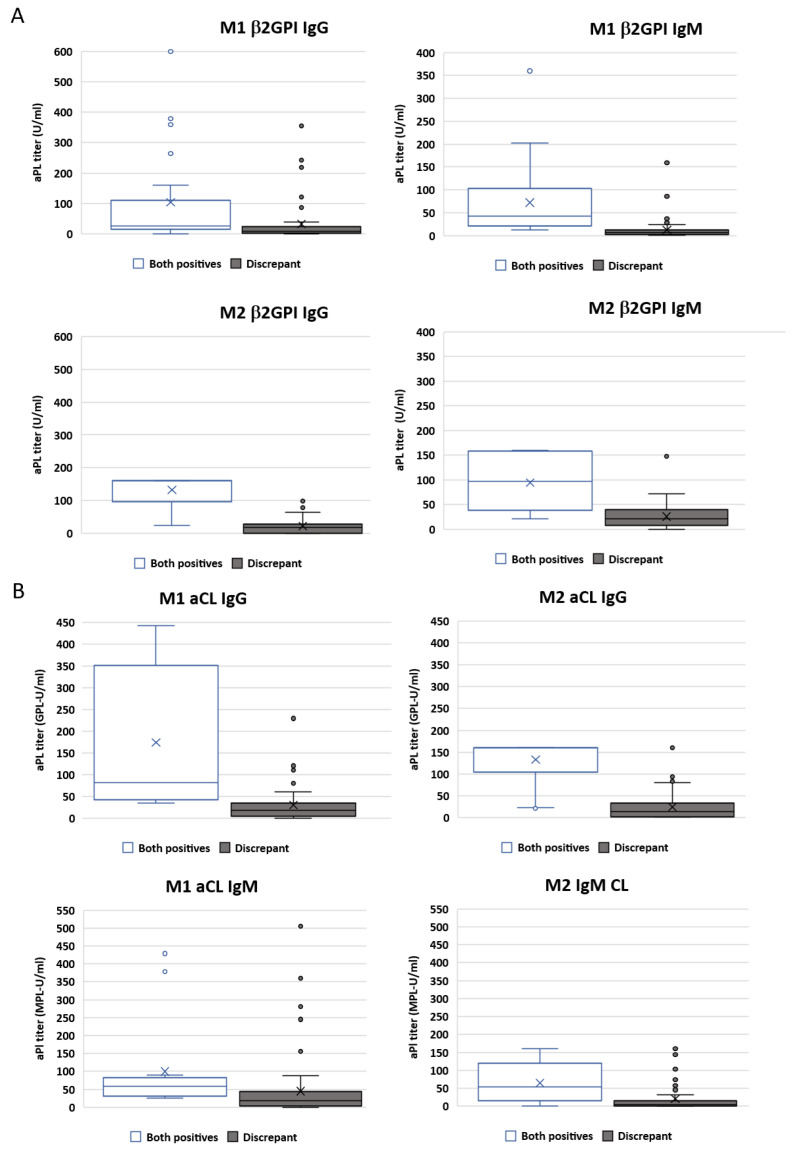
Antiphospholipid antibody (aPL) titres for samples in positive agreement and without agreement. (**A**) Titres for aβ2GPI IgG and IgM measured using M1 and M2. (**B**) Titres for aCL IgG and IgM measured using M1 and M2.

Among the 1020 patients analysed, we divided two patient groups for quantitative analysis: those displaying positive results for both methods and those with discrepant results. Samples that tested positive for aPLs only for one of the methods exhibited lower median aPL titres compared to those that showed agreement, with the majority of them falling within the near cut-off zone. In the opposite scenario, patients positive for both techniques presented higher titres of aPLs much further from the cut-off zone. For positive agreement samples and aβ2GPI isotypes, median values were higher for M2. The opposite trend was observed in the case of aCLs (Figure 3).

Moreover, in selecting those patients positive for any aPLs with high titres (three times higher than the cut-off), we did not find any additional correlation with APS events. (*p* = 0.475 for M1 and 0.083 for M2).

### 2.8. Diagnostic Performance

Globally, for the diagnosis of clinical events related to APS, the sensitivity obtained for M1 and M2 was 17.1% and 12.7%, respectively. Specificity values were similar: 89.3% for M1 and 91.4% for M2.

For M1, valid ROC curves were only obtained when considering obstetric morbidity patients, while, for M2, valid ROC curves were obtained only for patients with thrombotic events. However, AUC values were below 0.6 in all cases. (Table 7).

For thrombotic events, M1 had a sensitivity of 15.50% and a specificity of 87.84%, whereas M2 had a sensitivity of 13.07% and a specificity of 91.17%. In relation to obstetric morbidity associated with APS, only M1 showed a significant AUC with a *p*-value of 0.02, while M2 had a non-significant *p*-value of 0.49. The sensitivity and specificity for M1 were 23.75% and 87.66%, respectively. For M2, sensitivity and specificity were 12.50% and 90%, respectively. The most notable discrepancy was observed in patients with previous diagnoses of an autoimmune disease and obstetric events. Within this specific patient subgroup, the sensitivity of M1 was 50%, whereas M2 had half the sensitivity at 25% (Table 8).

Moreover, in patients with obstetric events, M1 presented higher positive likelihood ratios than M2, particularly in those with a previous diagnosis of an autoimmune disease (3.42 vs. 1.71). In contrast, for patients with thrombotic events, M2 displayed higher positive likelihood ratios.

The two methods exhibited a high degree of similarity for negative likelihood ratios, with the exception being patients with autoimmune disease and obstetric events. In these patients, the negative likelihood ratio was 0.59 for M1 and 0.88 for M2 (Table 9).

When considering the different aPLs, all sensitivities and specificities were consistently higher for IgG than for IgM, except for aβ2GPI detected by M1. Interestingly, this antibody demonstrated a higher specificity for IgM than for IgG. Moreover, aβ2GPI IgG detected by M1 had a higher sensitivity and positive likelihood ratio. In contrast, aCL IgM showed the worst sensitivity and positive likelihood ratio. Notably, the specificity and negative likelihood ratios were quite similar when considering all the aPLs for both testing methods (Table 10).

## 3. Discussion

Our analysis revealed that 70.27% of patients who were positive for at least one aPL were detected by only one of the two evaluated methods. Upon individual examination of each type of aPL, fair and moderate agreement between both methods was found for aCLs and aβ2GPI, respectively. Furthermore, it was identified that aCL IgM exhibited the poorest correlation between the analysed methods. Moreover, the results were affected by the clinical context. Interestingly, patients with a previous diagnosis of an autoimmune disease presented better concordance than those without previous autoimmunity towards other organs or systems. In patients with obstetric manifestations, M2 proved to be inadequate in assessing the risk of the patients, with only half the sensitivity of M1.

The presence of aPLs is significant for APS classification because they are key biomarkers used to establish appropriate therapies in patients with associated clinical manifestations. The variability of the results observed among the different commercially available methods as well as the need to improve harmonization and standardization have been previously reported [10,20]; this represents one of the most important challenges for accurate APS patient classification. The root cause of differences in test performance is typically due to test design, antigen characteristics and the polyclonal responses to diverse phospholipid antigens [21,22]. The incorporation of antigens into solid phase assays typically occurs through passive means, such as adhesion to the plastic substrate. In the case of bead-based assays, the process of incorporating antigens into microparticles is more complex, including processes such as avidin-mediated capture, which can be aggressive and potentially modify antigenicity by hiding epitopes or altering molecular conformation.

To our knowledge, our study includes one of the largest cohorts of patients that would normally receive referrals for aPL clinical routine testing. The cohort included 1020 patients, with 403 of them displaying clinical features compatible with APS. We also considered previous clinical events, accounting for 329 patients with prior thrombosis and 80 females who experienced pregnancy loss. Within our cohort, venous thrombosis was the most common event observed. This finding aligns with previous studies that have shown venous thrombosis as the predominant event in patients with APS [23,24,25,26].

Our results showed a generally poor correlation between the evaluated methods, with an overall kappa index of 0.38 and 0.36 when only patients with associated APS events were analysed. The lack of agreement observed had been documented previously; however, the agreement obtained was even lower in our study [10,20]. In addition, despite the size of our cohort, the results may differ depending on the population selected, for geographical region, gender or the prevalence of other autoimmune diseases.

Taking all patients into consideration (*n* = 1020), aβ2GPI IgG showed the best correlation, although it was only moderate (κ = 0.48) and aCL IgM the worst (κ = 0.28); this is in accordance with findings reported by other authors [12]. The inconsistency between methods found for aCL IgM was already mentioned in those publications, and it may also be in relation to a lower correlation with clinical manifestations and predictive value compared to IgG antibodies [27].

M1 displayed more isolated positives (for only one aPL) than M2, and M2 displayed more simultaneous positivity for both aPLs (aCLs and aβ2GPI). Notably, we found 32 patients who exhibited positivity for both aPLs using both methods. Regarding aCLs, it is noteworthy that there were more positive results obtained through each individual method (56 for M1 and 54 for M2) compared to the number of positive results achieved from both methods combined (41). Furthermore, aCLs demonstrated a higher detection rate than aβ2GPI.

The correlation coefficient was higher for patients with clinical APS manifestations, with no significant differences observed between patients with gestational or thrombotic APS. Our analysis also revealed that the total kappa index was notably higher for obstetric APS in comparison to thrombotic (0.46 vs. 0.33). This finding supports the idea that both manifestations of APS should be considered distinct entities, even when conducting aPL testing; this was also suggested by Chayoua et al., who proposed the use of two different decision trees for thrombosis and pregnancy morbidity. Specifically, they recommended testing for aCL and aβ2GPI IgM in women suspected of obstetric APS, while excluding those tests for patients suspected of thrombotic APS [28]. For obstetric APS, M2 identified a lower number of positive patients than M1 (11 vs. 2), suggesting lower sensitivity for M2. The obstetric aspects of APS have been discussed in various publications, which have highlighted the significance of low titre or single positive antiphospholipid antibodies (aPLs) in pregnancy-related complications. This observation probably contributes to the higher kappa index [29,30]. These findings support the recommendations of the new EULAR classification criteria to use solid phase assays instead of bead-based methods to determine aPLs [31], as is the case for M1. Therefore, the choice of methods based on solid phase is especially useful to correctly diagnose patients with obstetric APS.

Our results clearly showed that method concordance/agreement strongly depends on patient cohort selection and the specific aPL. Thus, thrombotic and obstetric APS should probably be diagnosed using two different algorithms, with the aPL serologic classification criteria for each being different.

The best correlation between techniques was achieved when considering patients with a previous diagnosis of an autoimmune disease (κ = 0.53). This finding may be associated with the over-representation of these specific patients in the standardization studies of the techniques. Furthermore, it is plausible that individuals with autoimmune disorders undergo aPL testing despite the absence of APS-related symptoms. The patients with the best agreement were those with previous autoimmune diseases and APS-associated clinical events. These findings are in line with the conclusions of the PRECISESADS study [32], which suggested that aPLs can be detected in other SARDS, and that this phenomenon is associated with clinical symptoms, including thrombosis and miscarriage.

Overall, we would like to highlight that the results from the most discrepant samples were near the cut-off value range, with a significant number of samples whose results were within that range. The low positive discrepancies between techniques may be clinically relevant for clinicians since the presence of the antibodies is considered for long- term treatment decisions [33]. This suggests that the definition of grey zones (equivocal ranges) by manufacturers is important and may contribute to harmonization efforts. In the absence of grey zones, patients who exhibit values near the cut-off point for aPL should undergo testing with an alternative method for more accurate evaluation, as previously suggested [34,35]. Given the absence of a gold standard method and the reliance on antibody positivity for the diagnosis of APS, it is very difficult to assess the superiority of one test over another. However, our results show that using methods with a defined grey zone can be more helpful. Despite the disagreement, both methods demonstrated an association with clinical events.

M2 showed a higher AUC in patients with thrombotic events, while M1 performed better in patients with obstetric APS. Valid ROC curves were obtained only for M1 when considering patients with gestational morbidity. The sensitivity was higher for M1 while the specificity was slightly higher for M2, suggesting that M1 is better for screening while M2 may be useful for result confirmation. The difference in sensitivity (and positive likelihood ratio) is especially notable in patients with gestational events and even more so in patients with prior autoimmune disease (50% vs. 25%). This suggests that M1 could be especially suitable for identifying obstetric APS. Furthermore, individuals with a previous autoimmune disease diagnosis exhibited a higher kappa index and sensitivity for both techniques. When examining each antibody, our findings indicate that IgG antibodies demonstrate greater sensitivity and specificity compared to IgM antibodies. This divergence in diagnostic performance may be in line with the recently published ACR/EULAR classification criteria for APS, which advocates higher importance for IgG antibodies [31].

Considering the aforementioned comments about current APS testing techniques, it must be remembered that APS is a life-threatening condition and huge efforts in standardization and reproducibility between methods are needed [22,36,37]. The advent of new diagnostic platforms [38,39] will presumably increase detection variability. Meanwhile, the use of two diagnostic methods may improve the diagnostic capacity of laboratories. Our results suggest that M1 and M2 can complement each other for adequate detection of patients with aPL and APS clinical manifestations.

Our study has some limitations. Firstly, it is crucial to acknowledge that the presence of aPLs is just one of several factors that can contribute to the occurrence of thrombosis or a miscarriage. Furthermore, there is a small subset of patients who do not exhibit the classical serological criteria but still test positive for non-Sidney aPLs. All of these factors introduce a bias into our evaluation, which currently poses a challenge, due to the absence of a universally accepted gold standard for comparison.

## 4. Materials and Methods

### 4.1. Study Design

This was an observational retrospective study of 1020 patients referred from specialist care due to the suspicion of APS. aβ2GPI and aCL antibodies were tested in the Immunology Service Laboratory, Hospital 12 de Octubre, from January 2019 to November 2020. APS suspicion was based on the following criteria: clinical symptoms (as per the classification criteria [40] and/or the presence of LA (lupus anticoagulant) positivity. Data on prior thrombotic events, miscarriages or other APS clinical manifestations in accordance with the classification criteria were gathered from the clinical records.

Patients were classified based on the existence of a previous clinical manifestation included in the APS Sidney classification criteria [40] into 3 groups: those with thrombotic manifestations, those with obstetric morbidity and those without clinical symptoms.

Moreover, we classified the cohort of patients based on the background of autoimmune diseases. For this study, we have only considered systemic autoimmune diseases, such as systemic lupus erythematosus (SLE), rheumatoid arthritis (RA), systemic sclerosis (SScl) and Sjogren’s syndrome (SS).

### 4.2. Cut-Off Established Using Healthy Donors

To establish the cut-off, we determined the p99 for each assay included in this study, using a cohort of 230 healthy donors following the Sydney classification criteria recommendations [40,41].

### 4.3. Assays

The aCL IgG, aCL IgM, aβ2GPI IgG and aβ2GPI IgM aPLs were measured at the Hospital Universitario 12 de Octubre using two commercially available automated assays. The first method (M1) was EliA^TM^ (Thermo Fisher Scientific, Phadia GmbH, Freiburg, Germany), a pure solid phase assay based on a fluorescence enzyme-immunoassay, carried out on a Phadia 250 instrument (Thermo Fisher Scientific, Phadia GmbH, Freiburg, Germany). The second assay (M2) was a laser bead-based, semi-solid phase immunoassay (Albia, BioPlex2200, Bio-Rad Laboratories, Hercules, CA, USA). All analytes were determined as per the specifications provided by the manufacturers using the obtained cut-off, as detailed above. For M1, the obtained p99 for aCLs were 18.5 GPL-U/mL for IgG, 22 MPL-U/mL for IgM and 11 U/mL for both aβ2GPI isotypes. For M2, the obtained threshold was 18 U/mL for aβ2GPI and aCLs for both isotypes.

LA was measured using two methods, HemosIL dRVVT (cutoff ratio 1.20) and HemosIL Silica Clotting Time (cutoff ratio 1.30) assays (Instrumentation Laboratory SpA, Milano, Italy) according to the guidance of the International Society on Thrombosis and Haemostasis [36].

### 4.4. Statistical Methods

The clinical and analytical information of the individuals was integrated into an anonymized database with no identifying references.

The sensitivity and specificity obtained by the area under the curve (AUC) were compared to calculate the diagnostic performance of the tests. The extent of concordance between the methods was evaluated using Cohen’s kappa index. To categorize the kappa index results, the scale proposed by Landis and Koch was used, adjusted to the five levels of the scale, based on the intensity of the association [19]. A kappa index from 0 to 0.20 was defined as slight, between 0.20 and 0.40 as fair, 0.40 and 0.60 as moderate, 0.6 and 0.8 as substantial and above 0.8 as nearly perfect. Comparison between the kappa index was performed using χ^2^. Data were analysed with MedCalc for Windows version 19.8 (MedCalc Software, Ostend, Belgium).

## 5. Conclusions

APS is a life-threatening condition defined by the presence of aPLs; therefore, the agreement between aPL testing methods is very important. To achieve this objective, we propose three keys: the inclusion of grey zones near the cut-off for determinations; the design of different testing algorithms for obstetric and thrombotic entities; and the use of an alternative confirmatory test to improve diagnosis approaches. Finally, selection of the technique could be important depending on the APS clinical manifestations, particularly when obstetric APS is suspected.

## Figures and Tables

**Table 1:** Demographic and clinical characteristics of included patients.

	Study Population (*n* = 1020)	
Variables	Median (SD)/*n*	IQR/%
Age	56 (19.13)	41–70
Sex (Male)	398	39.0
Dyslipidemia	247	24.2
Diabetes mellitus	151	14.8
Smoking	239	23.4
High blood pressure	324	31.8
Clinical manifestations included in APS classification criteria	403	39.5
Thrombotic events	329	81.64
Obstetric events	80	19.85
Autoimmune diseases	172	16.9
SLE	64	37.2
RA	36	20.9
SS	23	13.4
SScl	20	11.6

Abbreviations: SLE, systemic lupus erythematosus; RA, rheumatoid arthritis; SS, Sjögren syndrome; SScl, systemic sclerosis.

**Table 3:** Correlation between enzyme-linked immunosorbent fluorescent assay and addressable laser bead immunoassay.in patients with and without antiphospholipid syndrome events.

	APS Events (*n* = 403)		No APS Events (*n* = 617)	
aPL	Correlation Coefficient	*p*-Value	Correlation Coefficient	*p*-Value
aβ2GPI IgM	0.80	<0.0001	0.64	<0.0001
aCL IgM	0.46	<0.0001	0.18	<0.0001
aβ2GPI IgG	0.61	<0.0001	0.37	<0.0001
aCL IgG	0.71	<0.0001	0.49	<0.0001

Abbreviations: aPL: antiphospholipid antibody, aβ2GPI: anti-β-2 glycoprotein I autoantibodies I, aCL: anti-cardiolipin autoantibodies.

**Table 4:** Correlation between enzyme-linked immunosorbent fluorescent assay and addressable laser bead immunoassay in patients with thrombotic and gestational events.

	Thrombotic Events (*n* = 329)		Gestational Events (*n* = 80)	
aPL	Correlation Coefficient	*p*-Value	Correlation Coefficient	*p*-Value
aβ2GPI IgM	0.84	<0.0001	0.81	<0.0001
aCL IgM	0.50	<0.0001	0.48	<0.0001
aβ2GPI IgG	0.60	<0.0001	0.66	<0.0001
aCL IgG	0.70	<0.0001	0.66	<0.0001

Abbreviations: aPL: antiphospholipid antibody, aβ2GPI: anti-β-2 glycoprotein I autoantibodies I, aCL: anti-cardiolipin autoantibodies.

**Table 6:** Kappa index obtained in the different study groups.

	Autoimmune Disease	Non-Autoimmune Disease	*p*-Value
APS related event	0.55 (0.28–0.83)	0.30 (0.17–0.44)	0.110
Non-APS related event	0.55 (0.32–0.77)	0.37 (0.23–0.20)	0.182

Abbreviations: APS: antiphospholipid syndrome.

**Table 7:** Diagnostic accuracy of M1 and M2 methods for antiphospholipid syndrome patients with thrombotic and obstetric morbidity.

	AUC
M1 and thrombotic events	Non-significant (*p* = 0.16)
M2 and thrombotic events	0.52 (0.5–0.54)
M1 and obstetric morbidity considering only females	0.57 (0.52–0.62)
M2 and obstetric morbidity considering only females	Non-significant (*p* = 0.49)

Abbreviations: AUC: Area under the curve, M1: enzyme-linked immunosorbent fluorescent assay; M2, addressable laser bead immunoassay.

**Table 8:** Sensitivity and specificity of M1 and M2, considering autoimmune disease background and clinical events.

		M1		M2	
		Sensitivity	Specificity	Sensitivity	Specificity
All patients	APS event	17.12 (13.57–21.16)	89.30 (86.59–91.63)	12.66 (9.57–16.30)	91.41 (88.91–93.50)
	Thrombotic event	15.50 (11.76–19.87)	87.84 (85.17–90.19)	13.07 (9.62–17.20)	91.17 (88.80–93.18)
	Obstetric event	23.75 (14.95–34.58)	87.66 (85.39–89.69)	12.50 (6.16–21.79)	90.00 (87.90–91.84)
Autoimmune disease	APS event	27.66 (15.62–42.64)	88.00 (80.98–93.13)	23.40 (12.30–38.03)	88.80 (81.98–93.73)
	Thrombotic event	22.50 (10.84–38.45)	85.61 (78.44–91.11)	22.50 (10.84–38.45)	87.88 (81.14–92.87)
	Obstetric event	50.00 (17.70–84.30)	85.37 (79.01–90.39)	25.00 (3.19–65.09)	85.98 (79.70–90.90)
No autoimmune disease	APS event	15.73 (12.11–19.94)	89.63 (86.60–92.18)	11.24 (8.15–14.98)	92.07 (89.32–94.30
	Thrombotic event	14.53 (10.68–19.13)	88.37 (85.42–90.91)	11.76 (8.29–16.05)	91.95 (89.38–94.07)
	Obstetric event	20.83 (12.16–32.02)	88.14 (85.66–90.33)	11.11 (4.92–20.72)	90.85 (88.60–92.79)

Abbreviations: APS: antiphospholipid syndrome, M1: enzyme-linked immunosorbent fluorescent assay, M2: addressable laser bead immunoassay.

**Table 9:** Likelihood ratio considering autoimmune disease background and clinical events.

		M1		M2	
		Positive Likelihood Ratio	Negative Likelihood Ratio	Positive Likelihood Ratio	Negative Likelihood Ratio
All patients	APS event	1.6 (1.17–2.19)	0.93 (0.88–0.98)	1.47 (1.02–2.12)	0.96 (0.91–1)
	Thrombotic event	1.28 (0.92–1.76)	0.96 (0.91–1.02)	1.48 (1.03–2.14)	0.95 (0.91–1)
	Obstetric event	1.92 (1.25–2.95)	0.87 (0.77–0.99)	1.25 (0.68–2.30)	0.97 (0.89–1.06)
Autoimmune disease	APS event	2.3 (1.19–4.47)	0.82 (0.68–0.99)	1.95 (0.97–3.94)	0.87 (0.73–1.03)
	Thrombotic event	1.56 (0.77–3.18)	0.91 (0.76–1.08)	1.75 (0.84–3.61)	0.89 (0.74–1.06)
	Obstetric event	3.42 (1.56–7.49)	0.59 (0.29–1.17)	1.71 (0.49–6)	0.88 (0.59–1.32)
No autoimmune disease	APS event	1.52 (1.07–2.16)	0.94 (0.89–0.99)	1.42 (0.93–2.16)	0.96 (0.92.1.01)
	Thrombotic event	1.25 (0.87–1.79)	0.97 (0.91–1.02)	1.46 (0.96–2.23)	0.96 (0.91–1.01)
	Obstetric event	1.76 (1.08–2.87)	0.9 (0.8–1.01)	1.21 (0.61–2.42)	0.98 (0.9–1.06)

Abbreviations: APS: antiphospholipid syndrome, M1: enzyme-linked immunosorbent fluorescent assay, M2: addressable laser bead immunoassay.

**Table 10:** Sensitivity, specificity and likelihood ratio in different antiphospholipid antibodies.

		Sensitivity	Specificity	Positive LR	Negative LR
aCL M1	IgM	5.46 (3.5–8.2)	94.9 (92.9–96.6)	1.09 (0.6–1.9)	1.00 (0.97–1.03)
	IgG	7.94 (5.5–11.0)	96.11 (94.3–97.5)	2.04 (1.2–3.4)	0.96 (0.93–0.99)
aβ2GPI M1	IgM	5.71 (3.7–8.4)	97.08 (95.4–98.3)	1.96 (1.1–3.6)	0.97 (0.94–1.00)
	IgG	8.44 (5.9–11.6)	96.92 (95.2–98.1)	2.74 (1.6–4.7)	0.94 (0.91–0.98)
aCL M2	IgM	7.44 (5.1–10.5)	95.5 (93.5–96.9)	1.64 (1.0–2.7)	0.97 (0.94–1.00)
	IgG	7.69 (5.3–10.7)	96.11 (94.3–97.5)	1.98 (1.2–3.3)	0.96 (0.93–0.99)
aβ2GPI M2	IgM	6.70 (4.5–9.6)	94.81 (92.8–96.4)	1.29 (0.8–2.1)	0.98 (0.95–1.02)
	IgG	6.95 (4.7–9.9)	95.3 (93.3–96.8)	1.48 (0.9–2.5)	0.98 (0.95–1.01)

Abbreviations: LR: likelihood ratio, M1: enzyme-linked immunosorbent fluorescent assay, M2: addressable laser bead immunoassay, aCL: anti-cardiolipin autoantibodies, aβ2GPI: anti-β-2 glycoprotein I autoantibodies I.

## Data Availability

The data that support the findings of this study are available from the corresponding author upon reasonable request.
